# Evaluation of electrocardiographic alterations before and after bariatric surgery

**DOI:** 10.21542/gcsp.2025.22

**Published:** 2025-05-15

**Authors:** Shokoufeh Hajsadeghi, Aida Iranpour, Faranak Olamaeian, Ali Tayebi, Mahdis Gheitasi

**Affiliations:** 1 Research Center for Prevention of Cardiovascular Disease, Institute of Endocrinology and Metabolism, Iran University of Medical Sciences, Tehran, Iran; 2 Firoozabadi Clinical Research Development Unit (FACRDU) School of Medicine, Iran University of Medical Sciences, Tehran, Iran; 3 Department of Medicine, School of Medicine, Iran University of Medical Sciences, Tehran, Iran

## Abstract

**Background:** Obesity is a key risk factor for cardiac arrhythmia and sudden cardiac death. Additionally, morbid obesity is associated with an acquired elongation of the corrected QT interval, which could potentially lead to dangerous arrhythmias. In this study, we compare electrocardiography changes in patients with body mass index ≥40 before and after bariatric surgery.

**Methods:** We enrolled 55 patients with severe obesity in this study. All patients underwent bariatric surgery; electrocardiography along with anthropometric data and laboratory tests before and at least six months after bariatric surgery were performed.

**Results:** In total, 55 patients completed the study protocol (44 women and 11 men) and the mean age was 37.0 ± 10.5 years. Mean body mass index decreased from 46.7 ± 6.1 to 32.5 ± 5.1 (*P* < 0.0001) and the average variables of waist circumference, systolic and diastolic blood pressure, fasting blood sugar and glycated hemoglobin before and at least six months after bariatric surgery were significantly decreased (*P* < 0.0001). The postoperative assessment revealed a considerable decrease in the heart rate (*P* < 0.0001) and the corrected QT interval (*P* < 0.003).

**Conclusion:** This study showed that in a select group of patients with severe obesity, the corrected QT interval was reduced at least 6 months following the procedure. Also, as we expected, we observed decrease in systolic and diastolic pressure, heart rate, fasting blood sugar, glycated hemoglobin, the body indices including body mass index and waist circumference notably.

## Introduction

Obesity is recognized as an independent risk factor for various cardiovascular diseases, such as hypertension, congestive heart failure and ischemic heart disease^1^.

Additionally, obesity has been identified as a risk factor for ventricular arrhythmias and sudden cardiac death^1,2^. The prevalence of obesity has been on the rise due to lifestyle changes, emphasizing the significance of addressing the severe health consequences associated with it. Based on the World Health Organization, there are 2 billion people with overweight criteria, including 650 million adults with obesity. If these rates fail to decelerate, it is anticipated that by the year 2025, 2.7 billion adults will be overweight and 1 billion will be obese. The global prevalence of obesity in adults increased from 5% to 10.1% between 1980 and 2015 in men, while in women, it rose from 8.9% to 14.8% during the same period^3^. Given these alarming statistics, it is crucial to thoroughly examine the adverse effects resulting from severe obesity.

The electrocardiogram (ECG) is a readily available and useful tool for the initial evaluation of patients with cardiovascular conditions, providing valuable insights. Left ventricular hypertrophy, as assessed by measures like the Cornell voltage-duration product^4^ and echocardiography, is frequently observed in both obesity and hypertension, especially when these two conditions coexist^5^.

Recent studies have shown a higher prevalence of a prolonged corrected QT (QTc) interval in individuals with obesity^6,7^. The QTc interval is an index of ventricular repolarization, and its prolongation indicates an increased risk of ventricular arrhythmias and sudden cardiac death^8^. The prolonged QTc interval also serves as an index of sympathetic system hyperactivity under various cardiac conditions^9^. Recent studies have revealed that sympathetic system hyperactivity increases in both obese individuals and those with high blood pressure, particularly when these conditions coexist^10^.

Furthermore, based on conducted studies, it has been reported that individuals with severe obesity exhibit an increased duration of P wave due to thickening and expansion of epicardial fat. Epicardial fat is a layer of fatty tissue naturally lying between the myocardial and endocardial layers of the heart^11^.

Additionally, in certain studies, such as the one conducted by Sanches and Pouwels in 2020^12^, it has been reported that severe obesity is associated with cardiac rhythm disorders, such as atrial fibrillation, thereby elevating the risk of its occurrence.

A variety of scholarly investigations have been conducted to examine the impacts of weight loss surgeries, specifically bariatric surgeries, on enhancing cardiac function and mitigating complications associated with obesity. An example of a study carried out by Pontiroli et al. (2004) revealed a notable reduction in the QTc interval and left ventricular hypertrophy in individuals with obesity after undergoing bariatric surgery^13^.

This study aims to provide a comprehensive analysis of ECG alterations in individuals with severe obesity in both pre-and post-bariatric surgery. The rationale for conducting this study is rooted in the lack of similar investigations conducted in our country, as well as the need to further elucidate the ECG changes associated with severe obesity.

## Methods

### Study design

A cross-sectional retrospective descriptive-analytical study was conducted between March 2016 and March 2020 to compare ECG changes in patients with obesity after undergoing bariatric surgery. At the outset of the study, the researchers obtained the required permissions from the Ethics Committee of the medical university and hospital authorities to gather diverse information from the patients involved.

### Inclusion/exclusion criteria

**Figure 1.**
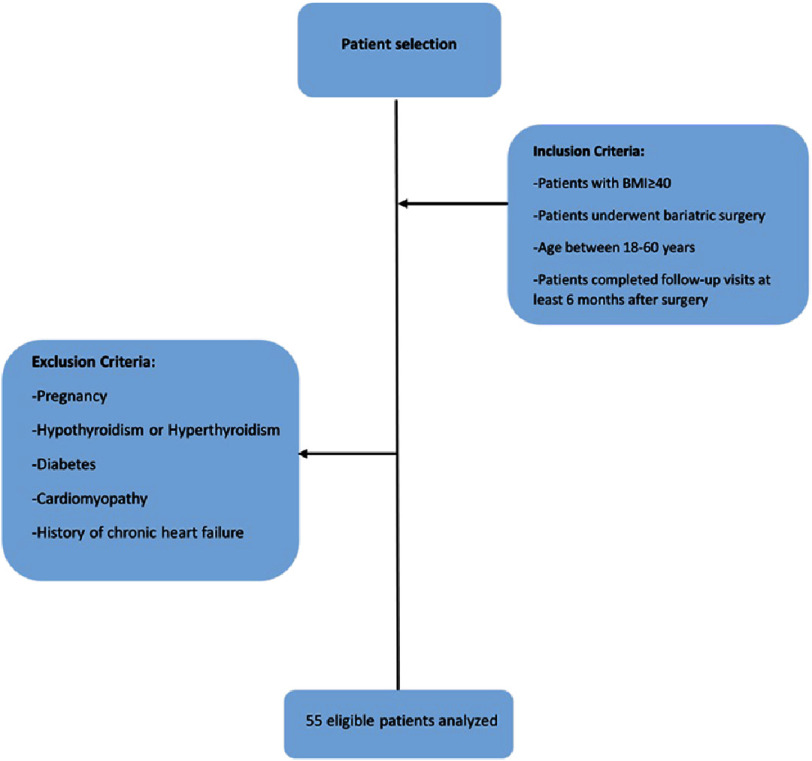
Flowchart for patient selection.

Patients with body mass index (BMI) ≥40, who underwent bariatric surgery, both those aged between 18 and 60 years old and those who had completed their follow-up visits at least 6 months after the surgery, were entered in the study. However, patients with pregnancy, Hypothyroidism or Hyperthyroidism, diabetes, cardiomyopathy, or a history of chronic heart failure were excluded (Figure 1).

### Sample size, preoperative assessment and EKG analysis

The sample size of the study includes 55 individuals who were classified as obese. We used G*Power 3.1.9.4 software to calculate the sample size, aiming for a power of 0.95 and an alpha of 0.05 based on prior studies. Before undergoing bariatric surgery, the patients underwent assessment before the procedure and at least six months post-surgery. The assessment primarily aimed to examine various demographic, clinical, and electrocardiographic variables. The researchers acquired twelve-lead ECG recordings at a rate of 25 mm/sec. The ECG analysis encompassed evaluations of various cardiac parameters, including rhythm, rate, axis, P wave morphology, PR interval duration, QT interval duration, presence of left ventricular hypertrophy, presence of right ventricular hypertrophy, presence of right bundle branch block, presence of left bundle branch block, presence of ST-T changes. The measurements of all ECG leads were reviewed manually. Furthermore, the QTc interval, which accounts for heart rate, was determined using Bazett’s equation: QTc = QT/√ (RR interval). Blood pressure and blood tests for fasting blood sugar (FBS) and glycated hemoglobin (HbA1c) were collected during the initial and subsequent ECG and echocardiographic examinations.

### Statistical analysis

In this study, continuous variables were characterized using statistical measures such as the mean and standard deviation, while categorical variables were expressed using numerical values or proportions. The Kolmogorov–Smirnov test was used to assess the normality of the data. Paired t-tests and Wilcoxon tests were utilized to conduct comparisons between continuous variables. The Chi-Square and Fisher exact tests were employed to examine the association between categorical variables. Significant differences were determined at a *P* value of less than 0.05. The statistical software utilized for the analyses was SPSS 26.

## Results

A total of 55 patients, consisting of 44 women and 11 men with a mean age of 37.8 ± 10.5 years (age 19–60 years), successfully completed the study protocol by undergoing bariatric surgery. Clinical and laboratory information is presented in Table 1. A significant reduction in the BMI was observed during the postoperative evaluation (*P* < 0.0001). After the completion of the surgical intervention, a significant reduction in waist circumference was observed (*P* < 0.0001). Furthermore, significant reductions were observed in both systolic blood pressure and diastolic blood pressure (*P* < 0.0001). There was also a notable enhancement in glycemic regulation, as evidenced by a decrease in FBS levels (*P* < 0.0001) and HbA1c levels (*p* < 0.006) within the examined cohort (Table 1).

**Table 1:** Clinical and laboratory data of subjects before and 6 months after bariatric surgery (Mean ± SD).

Data	Preoperative	Postoperative	*P*-values
Body mass index (BMI) (kg/m^2^)	46.7 ± 6.1	32.5 ± 5.1	0.0001
Waist circumference (cm)	119.0 ± 15.5	92.8 ± 9.7	0.0001
Systolic blood pressure (mmHg)	132.5 ± 14.2	120.3 ± 11.2	0.0001
Diastolic blood pressure (mmHg)	81.1 ± 9.5	70.7 ± 8.0	0.0001
FBS (mg/dl)	96.9 ± 12.1	87.4 ± 7.7	0.0001
HbA1c (Percent)	5.4 ± 0.4	5.1 ± 0.3	0.006

**Notes.** * Statistical analysis was conducted using a Paired T-tests and Wilcoxon tests, and P ¡ 0.05 was considered as a significant difference.

Table 2 displays electrocardiographic parameters. The postoperative evaluation revealed a significant decrease in heart rate (*P* < 0.0001) and QTc interval (*P* < 0.003). In contrast, an increase in the PR interval was observed; however, the difference was not statistically significant (Table 2 and Table 3).

**Table 2:** Data of subjects before and 6 months after bariatric surgery (Mean ± SD).

Data	Preoperative	Postoperative	*P*-values
PR interval (ms)	140.3 ± 25.3	143.2 ± 25.1	0.206
QTc (ms)	356.1 ± 42.3	341.9 ± 46.0	0.003
Heart rate (bpm)	79.8 ± 12.8	65.1 ± 8.40	0.0001

**Notes.** * Statistical analysis was conducted using a Wilcoxon tests, and *P* < 0.05 was considered as a significant difference.

**Table 3:** Electrocardiographic variables before and 6 months after bariatric surgery.

Data		Preoperative	Postoperative	*P*-values
Axis	Normal	54 (98.2%)	54 (98.2%)	1.000
	Right	1 (1.8%)	1 (1.8%)	
	Left	–	–	
P wave	Normal	55 (100%)	55 (100%)	1.000
	Abnormal	–	–	
Right Bundle Branch Block	Negative	54 (98.2%)	54 (98.2%)	1.000
	Positive	1 (1.8%)	1 (1.8%)	
Left Bundle Branch Block	Negative	55 (100%)	55 (100%)	1.000
	Positive	–	–	
Left Ventricular Hypertrophy	Negative	55 (100%)	54 (98.2%)	0.317
	Positive	–	1 (1.8%)	
Right Ventricular Hypertrophy	Negative	55 (100%)	55 (100%)	1.000
	Positive	–	–	
S-T Depression	Negative	55 (100%)	55 (100%)	1.000
	Positive	–	–	
S-T Elevation	Negative	54 (98.2%)	53 (96.4%)	1.000
	Positive	1 (1.8%)	2 (3.6%)	
T inversion	Negative	53 (96.4%)	50 (90.9%)	0.083
	Positive	2 (3.6%)	5 (9.1%)	

**Notes.** * Statistical analysis was conducted using a Fisher Exact test, and *P* < 0.05 was considered as a significant difference.

## Discussion

Obesity and overweight can lead to cardiovascular disorders as well as cause electrocardiographic disruptions. Bariatric surgery can play an important role in weight loss and improve electrocardiographic disruptions. This study aims to assess the impact of bariatric surgeries on clinical and laboratory markers, as well as electrocardiographic variables, within a sample of 55 individuals diagnosed with severe obesity. The Framingham Heart Study has reported that obesity is a robust indicator of sudden cardiac death on its own^14^.

Various factors may affect the development of malignant arrhythmias in individuals with obesity^15^. Currently, surface ECG serves as an uncomplicated and widely used tool that can be used for this purpose. The prolongation of the QTc interval, which serves as an indicator for electrical instability and sudden cardiac death, exhibited a notably greater magnitude in patients with obesity compared to individuals with a normal weight^16^. The majority of the existing data has revealed that reducing body weight in patients with obesity leads to a meaningful enhancement of the variables related to ventricular repolarization^17^. Grasser EK and co-workers studied the changes of the QTc interval in electrocardiograms in 49 patients with obesity before and 12 months after weight loss surgery. They reported a decrease in QTc interval duration by using four different QT correction equations, and this finding has also been confirmed by this current study^6^. Papaioannou A and co-workers also studied the effect of weight loss on the QTc interval duration in 17 patients with obesity before and 8–10 months after the surgery. They reported that significant weight loss was accompanied by a shortening of the QTc interval.

We used a greater sample of patients (55 patients) with the same result of a decreased QTc interval^18^. Sanches E and co-workers conducted a study to evaluate the impact of weight loss surgery on heart rhythm. They reported that QTc interval length and p wave duration decreased, but the impact on atrial fibrillation was not revealed. In our study, QTc interval duration also decreased, additionally, there was an increase in the PR interval; however, the difference was not statistically significant^12^. In the study conducted by Fernandes-Cardoso A and co-workers, the electrocardiograms changes in 20 patients with severe obesity (age: 36.3 ± 10.2) were evaluated before and 12 months after bariatric surgery. The writers documented a statistically significant reduction in both body mass index and P wave duration, attributed to the reduction in epicardial adipose.

In our study, we examined a larger sample of 55 severe patients with obesity (age: 37.8 ± 1) before and at least 6 months after bariatric surgery, and we observed a notable decrease in BMI without any alteration in either P wave duration or PR interval^19^. This non-significant increase in the PR interval and the lack of changes in the P wave could be due to potential mechanism, such as incomplete resolution of structural changes like atrial fibrosis or remodeling, alteration in autonomic tone (either parasympathetic or sympathetic activity), limitation in measurement techniques and relatively short follow-up period. Potential cofounders such as postoperative medications, physical activity levels, other treatment and genetic factors could have influenced the ECG parameters. Waleed Ammar and co-workers studied the cardiovascular outcomes after bariatric surgeries in patients with morbid obesity, and the investigation evaluated a sample of 100 patients compared to our sample of 55 patients. Both studies evaluated the characteristics of patients who underwent surgery at least 6 months following the procedure. Additionally, the prior study had a greater number of female subjects, 84% of the overall population, whereas in our study, the female content was 80%. In both studies, several findings were noted, including a significant reduction in BMI, heart rate, systolic and diastolic pressure, as well as FBS and QTc duration, at least 6 months following the bariatric surgery^20^.

## Limitations

This study has some key limitations, including the lack of a control group and a small sample size, which may have impacted the results. Further studies with larger sample sizes and control groups are needed to confirm these findings.

## Conclusion

Our study findings indicate that among individuals with severe obesity who have undergone bariatric surgery, there is a notable reduction in the QTc interval at least 6 months after surgery. This reduction is strongly correlated with the significant weight loss resulting from the surgical interventions. Additionally, a significant decrease in systolic and diastolic blood pressure, heart rate, FBS levels, HbA1c levels, and various body indices such as BMI and waist circumference were observed. However, further studies are needed to understand whether the weight loss associated with QTc reduction, decreases the risk of cardiac arrhythmia and sudden cardiac death in obesity.

## Author Contributions

**Conceptualization:** Shokoufeh Hajsadeghi, Aida Iranpour, and Faranak Olamaeian.

**Data curation:** Mahdis Gheitasi.

**Formal analysis:** Mahdis Gheitasi.

**Supervision:** Shokoufeh Hajsadeghi, Aida Iranpour, Faranak Olamaeian, and Ali Tayebi.

**Visualization:** Shokoufeh Hajsadeghi, Aida Iranpour, Faranak Olamaeian, Ali Tayebi and Mahdis Gheitasi.

**Writing - original draft:** Mahdis Gheitasi.

## Declarations of interest

The author(s) declared no potential conflicts of interest with respect to the research, authorship, and/or publication of this article.

## Funding

The author(s) received no financial support for the research, authorship, and/or publication of this article.

## Ethical approval

The project was approved by Ethical committee of Iran University of Medical Sciences (Ethical code: IR.IUMS.REC.1399.1317) on February 27, 2021.

Written informed consent was obtained from all participants in accordance with ethical standards and the study protocol approved by the ethics committee.
